# Effects of dietary supplementation with different concentration of molasses on growth performance, blood metabolites and rumen fermentation indices of Nubian goats

**DOI:** 10.1186/s12917-020-02636-5

**Published:** 2020-10-29

**Authors:** Osman A. Osman, Nawal M. Elkhair, Khalid A. Abdoun

**Affiliations:** 1 Department of Physiology, Faculty of Veterinary Science, University of West Kordofan, Al-Fulah, Sudan; 2 Department of Physiology, Faculty of Veterinary Medicine, University of Khartoum, 13314 Shambat, Sudan; 3 Department of Biomedical Sciences, College of Veterinary Medicine, King Faisal University, Al-Ahsa, 31982 Saudi Arabia; 4 Department of Animal Production, Faculty of Food and Agriculture Sciences, King Saud University, Riyadh, Saudi Arabia

**Keywords:** Blood metabolites, Goat kids, Growth performance, Molasses, Rumen fermentation

## Abstract

**Background:**

Molasses is a potential energy supplement; extensively used to improve growth performance, milk and meat characteristics in goats at relatively low concentrations of 5–40% of the diet. Few data are available concerning feeding molasses to goat kids; therefore, the current study aimed to investigate the effects of dietary supplementation with higher concentrations of molasses on growth performance, blood metabolites and rumen fermentation indices. Twenty male Nubian goat kids (4–6 months old; 9–10 kg BW) were randomly assigned to 4 groups receiving different concentration of molasses: 0% (M-0), 30% (M-30), 40% (M-40) and 45% (M-45) for 5 weeks. Feed (DFI) and water intake (DWI) were measured daily, while the blood and rumen liquor samples were collected weekly.

**Results:**

The DFI increased and feed conversion ratio (FCR) decreased in all molasses-supplemented groups (*P* ≤ 0.05), whereas DWI increased in M-30 and decreased in M-45 (P ≤ 0.05). The final BW and average daily gain (ADG) increased (*P* < 0.0001) in groups M-30 and M-40 compared to the control and M-45. Blood pH was significantly influenced by dietary molasses concentration (MC) and the duration of molasses supplementation (MD), where it decreased in groups M-30 and M-45 compared to the control and M-40 (*P* < 0.05). The MC had no significant effect on blood Hb, HCT, TLC, albumin, [K^**+**^], AST, ALT and total protozoa count (TPC), as well as ruminal-[Na^**+**^], [K^**+**^], strong ion difference concentration ([SID_3_]) and [NH_3_]; however, only [NH_3_] was significantly affected by MD and the interaction between MC and MD (MC × MD). Serum TP, globulins, [Na^**+**^] and [Cl^**−**^] increased (*P* ≤ 0.05) in all supplemented groups, while A/G ratio and [SID_3_] decreased (P ≤ 0.05). Ruminal pH decreased (*P* < 0.0001) in M-40 and M-45 compared to the control and M-30. However, [VFAs] increased (*P* < 0.04) in M-30 and M-40 compared to the control and M-45, while osmolality increased (*P* ≤ 0.05) in M-30 compared to the other groups.

**Conclusions:**

Dietary supplementation with molasses at a concentration of 30% for 3 weeks improved growth performance, protein metabolism and rumen fermentation without compromising animal health, immunity, and electrolytes and acid-base homeostasis.

## Background

Goats (*Capra hircus*) play an important and critical role in the communities’ livelihood in arid and semi-arid regions in addition to their contribution to human food security by producing milk and meat, and for providing leader and manure as a fertilizer [1, 2]. Therefore, goats are recommended livestock in tropics with minimal maintenance and low water requirements [3–5]. The total population of goats in Sudan is estimated to be 31.44 million heads [6], which are mainly distributed in all agro-ecological zones from arid Northern regions to humid Southern Sudan. Goats in Sudan are classified into four distinct and well-known ecotypes as Nubian, Desert, Nilotic, and Mountain goats [7]. The Nubian goats are widely distributed in arid and extreme arid areas in Northern Sudan [8], which are considered the main dairy breed among the other goat breeds.

In developing countries, industrial plants’ by-products are being commonly used as raw materials in ruminant diets due to the shortage of feedstuff for livestock combined with an increase in their price and restrictions [9]. In Sudan, molasses is an important by-product of the sugar industry [10]. Increasing demands for energy and protein by productive ruminants increased the importance of molasses as an energy supplement with a concentrated source of fermentable sugars and low protein content [11]. Molasses is extensively used in livestock feeding to improve palatability and to reduce dustiness [12]. Furthermore, molasses has been used for a long time as a carrier for urea and mineral supplements to provide a slow, continuous intake of nutrients required for an optimum environment for microbial fermentation [13, 14]. Therefore, valuable research has been conducted on feeding molasses to dairy and beef cattle [11, 15–17] and sheep [18–20]. Other researchers used urea-molasses mineral blocks or urea-molasses to improve growth and production performance in goats [21, 22]. However, few data are available with regard to feeding molasses to goat kids or to assess blood metabolites and rumen fermentation indices in response to dietary supplementation with molasses [13, 14, 22]. A study conducted by Babikir [23] concluded that dietary supplementation with 20% molasses improved feedlot performance of Nubian goat kids; however, the author did not study the effect of molasses on blood metabolites and rumen fermentation. Consequently, the current study hypothesized that further increase in the concentration of molasses supplementation might result in a better performance of goat kids without compromising their health status. Therefore, the present study aimed to evaluate the effects of dietary supplementation with higher concentrations of molasses on growth performance, blood metabolites and rumen fermentation of male Nubian goat kids reared under Sudan tropical conditions.

## Results

### Growth performance, daily feed and water intake

The results presented in Table 1 show that dietary supplementation with different concentration of molasses increased (*P* < 0.01) feed conversation ratio, daily feed intake and water intake (DFI and DWI). The highest final BW and ADG (*P* < 0.0001), and DWI (*P* = 0.02) was observed in group M-40 compared to the other groups.

**Table 1:** Effects of dietary supplementation with different concentrations of molasses on growth performance, and daily feed and water intake of male Nubian goat kids

Parameters	Groups				***P***- value		
	M-0	M-30	M-40	M-45	MC	MD	MC×MD
Initial BW (kg)	10.0 ± 0.8	10.3 ± 0.7	10.6 ± 0.7	9.6 ± 0.6	NS	**–**	**–**
Final BW (kg)	10.4 ^b^ ± 0.8	10.9 ^a^ ± 0.8	11.4 ^a^ ± 0.9	9.9 ^c^ ± 0.6	***	**–**	**–**
BWG (kg)	0.4 ^b^ ± 0.2	0.6 ^a^ ± 0.2	0.8 ^c^ ± 0.3	0.4 ^b^ ± 0.1	***	**–**	**–**
ADG (kg/day)	0.06 ^b^ ± 0.02	0.1 ^a^ ± 0.02	0.11^a^ ± 0.04	0.05 ^b^ ± 0.02	***	**–**	**–**
FCR	16.2 ^a^ ± 0.3	11.3 ^b^ ± 0.7	10.8 ^b^ ± 0.3	23.8 ^a^ ± 0.6	**	**–**	**–**
DFI (kg)	0.969 ^c^ ± 281	1.126 ^b^ ± 250	1.186 ^a^ ± 315	1.187 ^a^ ± 174	**	NS	NS
DWI (Litre)	1.298 ^b^ ± 492	1.153 ^b^ ± 363	1.605 ^a^ ± 575	0.981 ^c^ ± 273	***	*	NS

^a, b, c^ *Means ± SD within the same row bearing different superscripts are significantly different at* ^*^P < 0.05, ^**^P < 0.01, ^***^P < 0.0001

*NS* Not significant, *SD* Standard deviation, *BW* Body weight, *BWG* Body weight gain, *ADG* Average daily gain, *FCR* Feed conservation ratio, *DFI* Daily feed intake, *DWI* Daily water intake, *MC* molasses concentration, *MD* the duration of dietary molasses supplementation, *ML×MD* the interaction between molasses concentration and the duration of dietary molasses supplementation, *M-0* (0% molasses)*, M-30* (30% molasses)*, M-40* (40% molasses) and *M-45* (45% molasses)

### Blood and serum parameters

The results of dietary molasses supplementation on blood and serum biochemical parameters are presented in Table 2. The mean values of blood pH ranged between 7.33–7.37. Dietary increase concentration of molasses (MC) and the duration of dietary molasses (MD) supplementation decreased blood pH (*P* = 0.02) in supplemented groups M-30 and M-45 (7.33) compared to the control group and M-40 (7.34 and 7.37, respectively). Furthermore, blood pH decreased (*P* < 0.0001) with the duration of dietary molasses supplementation (Table 2).

**Table 2:** Effects of dietary supplementation with different concentrations of molasses on blood and serum biochemical variables of male Nubian goat kids

Parameters	Groups				***P***- value		
	M-0	M-30	M-40	M-45	MC	MD	MC×MD
Blood pH	7.34 ^**a**^ ± 0.06	7.33 ^**a**^ ± 0.05	7.37 ^**b**^ ± 0.05	7.33 ^**a**^ ± 0.05	*	***	NS
Blood-[Hb] (g/dL)	10**.**0 ± 1.2	9**.**0 ± 1.4	8.7 ± 1.1	8.9 ± 1.1	NS	NS	NS
Blood Hct (L/L)	0.26 ± 0.05	0.27 ± 0.03	0.28 ± 0.03	0.26 ± 0.03	NS	NS	NS
TLC (**×**10^3^/μL)	9.6 ± 2.9	9.3 ± 3.7	8.7 ± 2.9	9.0 ± 3.6	NS	NS	NS
Serum-[TP] (g/L)	61 ^**b**^ ± 4.1	63.6 ^**ab**^ ± 6.4	65.6 ^**a**^ ± 4.3	63.1 ^**ab**^ ± 10.6	*	*	NS
Serum-[Albumin] (g/L)	36.9 ± 3.7	37.6 ± 3.7	37.5 ± 3.5	37.1 ± 2.5	NS	***	NS
Serum-[Globulins] (g/L)	24 ^**b**^ ± 5.4	26 ^**ab**^ ± 5.8	28 ^**a**^ ± 4.6	26 ^**ab**^ ± 10.8	*	NS	NS
A/G (%)	1.6 ^**a**^ ± 0.6	1.5 ^**ab**^ ± 0.4	1.4 ^**ab**^ ± 0.3	1.2 ^**b**^ ± 0.7	*	NS	NS
Serum-[AST] (IU)	41.7 ± 27.5	43 ± 13	45 ± 6.3	42 ± 24.2	NS	NS	NS
Serum-[ALT] (IU)	11.7 ± 6.9	10.7 ± 3.7	10.9 ± 7.6	11 ± 8.2	NS	NS	*
Serum-[Na^+^] (mmol/L)	153.7 ^**b**^ ± 6.9	154.9 ^**b**^ ± 7.3	148.6 ^**a**^ ± 8	150.2 ^**b**^ ± 7.9	**	NS	NS
Serum-[K^+^] (mmol/L)	6.2 ± 1.1	5.7 ± 0.8	6.6 ± 1.5	6.0 ± 1.3	NS	***	**
Serum-[Cl^−^] (mmol/L)	106.8 ^**b**^ ± 5.8	111.3 ^**a**^ ± 6.0	105.7 ^**b**^ ± 5.3	106.9 ^**b**^ ± 5.5	**	NS	**
Serum-[SID_**3**_] (mmol/L)	53.1 ^**a**^ ± 6.6	49 ^**b**^ ± 7.1	49.4 ^**b**^ ± 7.5	49.3 ^**b**^ ± 6.3	*	NS	NS

^a, b^ *Means ± SD within the same row bearing different superscripts are significantly different at* ^*^P ≤ 0.05, ^**^P ≤ 0.01, ^***^P < 0.0001. NS Not significant

*Brackets ([ ]) donate concentration, SD* Standard deviation

*MC* molasses concentration, *MD* the duration of dietary molasses supplementation, *ML×MD* the interaction between molasses concentration and the duration of dietary molasses supplementation, *M-0* (0% molasses), *M-30* (30% molasses), *M-40* (40% molasses) and *M-45* (45% molasses)

The mean values of the hematological parameters ranged between 8.7–10 g/dL for Hb, 0.26–0.28 L/L for HCT, and 8.7–9.6 × 10^3^/μL for TLC; where dietary molasses supplementation did not show significant effects on Hb, Hct and TLC (Table 2).

### Serum protein parameters, AST and ALT

The mean values of serum proteins were 61–65.6 g/L for TP, 36.9–.37.6 g/L for albumin, 24–28 g/L for globulins, and 1.2–1.6 for A/G ratio. Serum TP and globulins increased (*P* ≤ 0.05) in all molasses supplemented groups (63–65.6 and 22–28 g/L, respectively) compared to the control group (61 and 24 g/L, respectively). More pronounced increase in TP (65.6 g/L, *P* = 0.05) and globulins (28 g/L, *P* = 0.03) was observed in the supplemented group M-40 compared to the other groups. The A/G ratio decreased (*P* = 0.02) in all supplemented groups (1.2–1.5) compared to the control group (1.6); the lowest value was observed in supplemented group M-45 (1.2). The MD had a significant influence (*P* < 0.0001) only on serum albumin concentration (Table 2).

The mean values of AST and ALT were 28–45 IU and 10.7–11 IU, respectively, where dietary molasses did not show significant effects on serum AST and ALT concentrations (Table 2). However, MC × MD had a significant influence (*P* = 0.05) on ALT.

### Serum electrolytes and SID_3_

**Fig. 1:**
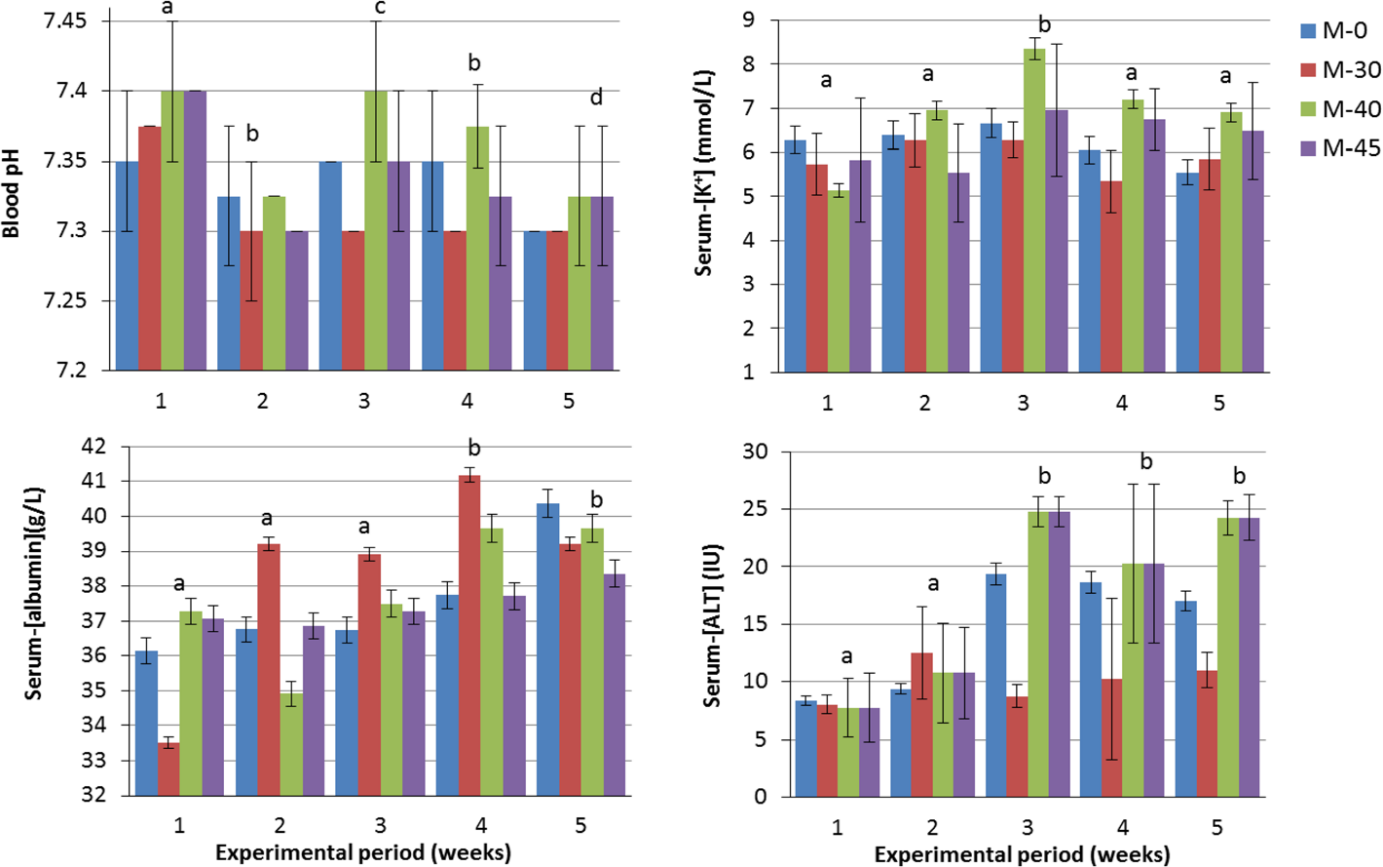
Changes in blood pH, serum-[K^+^], [albumin] and [ALT] of Nubian goat kids as influenced by the duration of dietary supplementation with different concentrations of molasses

The mean values of serum electrolytes and SID_3_ ranged between 148.6–154.9 mmol/L for Na^+^, 5.7–6.6 mmol/L for K^+^, 105.7–111.3 mmol/L for Cl^−^, and 49–53 mmol/L for SID_3_. Serum-[Na^**+**^] decreased (*P* = 0.006) in the supplemented group M-40 (148.6 mmol/L) compared to the control group (153.7 mmol/L) and M-30 (154.9 mmol/L). The MD and MC × MD had a significant effect on serum-[K^**+**^] (*P* = 0.0001 and 0.007, respectively), which increased in week 3 in supplemented groups M-40 and M-45 compared to the control group and M-30 (Fig. 1). Serum-[Cl^−^] increased (*P* ≤ 0.05) in the supplemented group M-30 (111 mmol/L) compared to the control group (106.8 mmol/L), M-40 (105.7 mmol/L), and M-45 (106.9 mmol/L). The interaction between MC × MD has a significant effect on [Cl^−^] (*P* = 0.01, Table 2). Serum-[SID_3_] decreased (*P* = 0.02) in all supplemented groups (49 mmol/L) compared to the control group (53 mmol/L).

### Rumen fermentation indices

The results of the effect of dietary supplementation with different concentration of molasses on rumen fermentation indices are presented in Table 3. The mean values were 6.3–6.48 for pH, 72.5–85.6 mmol/L for VFAs, 7.6–10.5 mg/dL for NH_3_, 24.5–26.8 × 10^4^/ml for TPC, 242–289 mOsmol/kg for osmolality, 154–158 mmol/l for Na^+^, 29.8–32.3 mmol/l for K^+^, 106.6–110.7 mmol/L for Cl^−^, and 75–82 mmol/L for SID_3_.

**Table 3:** Effects of dietary supplementation with different concentrations of molasses on rumen fermentation indices of male Nubian goat kids

Parameters	Groups				***P***- value		
	M-0	M-30	M-40	M-45	MC	MD	MC×MD
Ruminal pH	6.48 ^**a**^ ± 0.1	6.46 ^**a**^ ± 0.1	6.39 ^**b**^ ± 0.1	6.30 ^**b**^ ± 0.2	***	NS	NS
VFAs (mmol/L)	77.7 ^**b**^ ± 23	85.6 ^**a**^ ± 19	81.9 ^**a**^ ± 15	72.5 ^**b**^ ± 17.6	*	***	NS
NH_**3**_ (mg/dL)	8.7 ± 4.7	10.5 ± 5.1	8.8 ± 4.5	7.6 ± 4.2	NS	*	**
Total protozoa count (×10^**4**^/mL)	24.5 ± 4.5	26.8 ± 6.3	26.3 ± 4.9	26 ± 4.3	NS	NS	NS
Osmolality (mOsmol/kg)	260 ^**b**^ ± 38	289 ^**a**^ ± 27	248 ^**c**^ ± 35	242 ^**c**^ ± 38	***	NS	NS
Ruminal -[Na^+^] (mmol/L)	155 ± 9	158 ± 8	154 ± 11	154 ± 11	NS	NS	NS
Ruminal -[K^+^] (mmol/L)	29.8 ± 5.9	32.3 ± 5.9	31.8 ± 5	31.9 ± 6.1	NS	**	NS
Ruminal -[Cl^−^] (mmol/L)	106.6 ^**b**^ ± 4.8	108 ^**b**^ ± 3.8	110.7 ^**a**^ ± 4.7	109.6 ^**a**^ ± 4.6	**	NS	NS
Ruminal-[SID_**3**_] (mmol/L)	78.5 ± 14.6	82.2 ± 12	74.9 ± 12	75.7 ± 13	NS	NS	NS

^a, b, c^ *Means ± SD within the same raw bearing different superscripts are significantly different at* ^*^P ≤ 0.05, ^**^P ≤ 0.01, ^***^P < 0.0001. NS Not significant

*Brackets ([]) donate concentration, SD* Standard deviation

*MC* molasses concentration, *MD* the duration of dietary molasses supplementation, *ML×MD* the interaction between molasses level and the duration of dietary molasses supplementation, *M-0* (0% molasses)*, M-30* (30% molasses), *M-40* (40% molasses) and *M-45* (45% molasses)

Ruminal pH decreased (*P* = 0.0001) in the supplemented groups M-40 and M-45 (6.39 and 6.30, respectively) compared to the control and M-30 groups (6.48 and 6.46, respectively). The MC and MD had significant effect on VFAs (*P* = 0.04 and 0.0001, respectively), which increased significantly (*P =* 0.04) in the supplemented group M-30 (85.6 mmol/L) compared to the control (77.7 mmol/L) and M-45 (72.5 mmol/L).

The MC had no significant effect on ruminal NH_3_ (Table 3). However, MD and the interaction between MC × MD have significant effects on NH_3_ (*P* = 0.0001 and 0.007, respectively).

### Total protozoa count (TPC) and osmolality

Higher TPC was observed in all supplemented groups (26–26.8 × 10^4^/mL) compared to the control group (24.5 × 10^4^/mL); however, the increase was not statistically significant (*P* = 0.23).

Ruminal osmolality increased (*P =* 0.0001) in M-30 and decreased in M-45 compared to the control group; the more pronounced increase was observed in M-30 (289 mOsmol/kg) compared to the control, M-40 and M-45 groups (260, 248 and 242 mOsmol/kg, respectively).

### Ruminal electrolytes and SID_3_

**Fig. 2:**
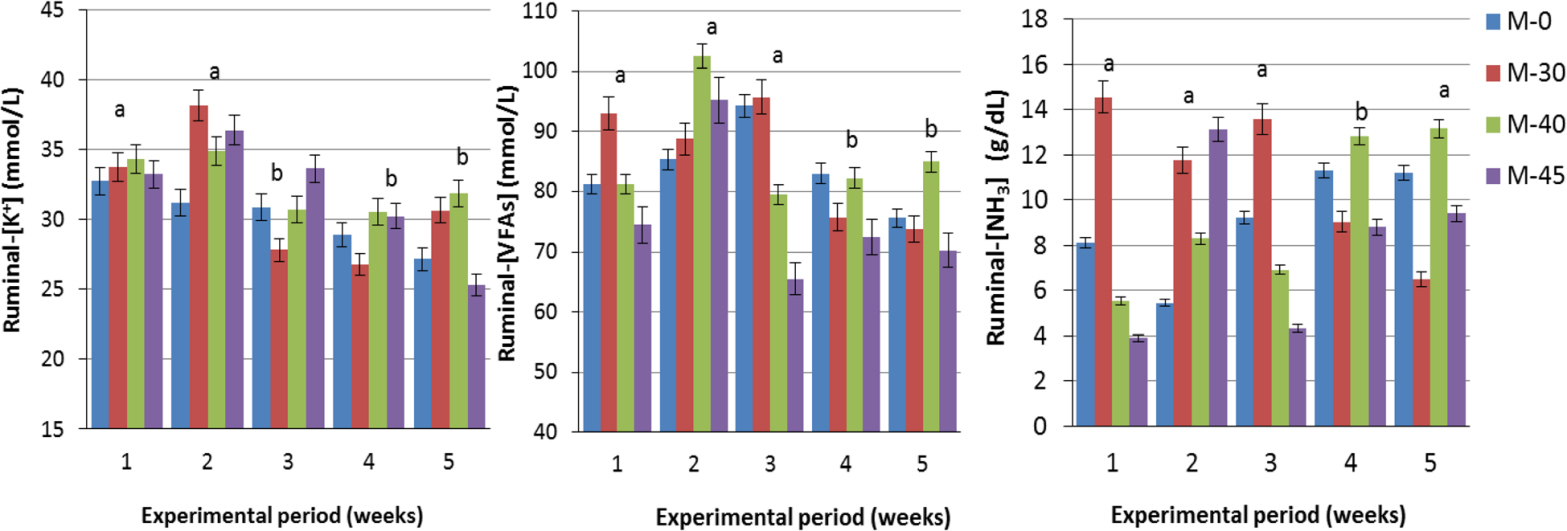
Changes in ruminal-[K^+^], [VFAs] and [NH_3_] of Nubian goat kids as influenced by the duration of dietary supplementation with different concentrations of molasses

The MC had no significant effects on ruminal [Na^**+**^] and [K^**+**^]; however, MD had a significant (*P* = 0.003) effect only on [K^**+**^], which increased in weeks 4 and 5 compared to weeks 1, 2 and 3 of molasses supplementation (Fig. 2).

Ruminal-[Cl^**−**^] increased (*P* = 0.005) in the supplemented groups M-40 and M-45 (110.7 and 109.6 mmol/L, respectively) compared to the control and M-30 groups (106.6 and 108 mmol/L, respectively); the highest value was observed in M-30 (Table 3). However, the ruminal-[SID_3_] tended to change insignificantly (*P* = 0.23) in response to dietary molasses.

## Discussion

The aim of this study was to assess the effects of dietary supplementation with different concentration of molasses on growth performance, blood metabolites and rumen fermentation indices in male Nubian goat kids. The main finding obtained in the present study was that dietary supplementation with molasses improved growth performance of Nubian goat kids as indicated by higher final BW and BWG, and lower FCR, without interfering with the immunological function and acid-base homeostasis.

### Growth performance

The results obtained in the present study showed that the supplemented groups M-30 and M-40 (30 and 40% of molasses) consumed more feed and water compared to the control group and M-45, which reflected positively on kids’ growth performance. The improvement in the final BW may be due to the increase in palatability caused by molasses. Many investigators concluded that the addition of molasses to animal feed improved growth performance in lambs [18], calves [15, 16] and beef steers [17]. Other researchers reported that urea-molasses-mineral blocks and 20% molasses improved growth and production performance in goats [22, 23].

### Blood and serum parameters

The results obtained in the present study showed that the mean values of blood pH for all groups (7.33–7.37) were lower than the normal pH for goats (7.36) [24], which can be explained as a physiological response to the changes in the environmental temperature because the experiment was conducted during summer season. In the present study, the pH decreased significantly with the duration of molasses supplementation (Fig. 1). The secondary drop in the blood pH (acidemia) after 3 weeks of molasses supplementation; can be considered as a logical result of the physiological adaptation to dietary changes. Furthermore, the significant decrease in the pH in all kids after 4–5 weeks of molasses supplementation could be attributed to the accelerated microbial activities as indicated by the observed increase in TPC.

In the present study, the hematological parameters such as Hb, HCT and TLC were not influenced by dietary molasses supplementation, where none of these parameters differed from those of the control group. All hematological values were within the normal ranges for goats [25–27] suggesting that molasses supplementation had no adverse effects on both the hematopoietic and immune systems. Similar results have been reported in Boer and local Mahari goats fed molasses/mineral blocks [14, 22] and in West African Dwarf goats fed cassava peels with urea-molasses multi-nutrient block supplements [28].

The mean values for serum TP, albumin and globulins obtained in the present study were within the normal ranges for goats [25]. The significant increase in TP and globulins in all molasses supplemented groups is explained by the improved feed utilization as indicated by the observed significant decrease in FCR (Table 1). On the other hand, the significant increase in globulins accompanied by a decrease in A/G ratio in all molasses supplemented groups implies that molasses supplementation might enhance the humoral immunity response in Nubian goat kids. The improvement in serum protein parameters observed in the present study was in agreement with previous reports in Boer and local Mahari goats fed molasses/mineral blocks [14, 22] and in West African Dwarf goats fed cassava peels with urea-molasses multi-nutrient block supplements [28]. Additionally, it has been concluded that dietary supplementation with urea-molasses mineral granules had no significant effect on albumin concentration in goat kids [13].

The results obtained in the present study showed that serum AST and ALT were not changed significantly in response to dietary molasses supplementation. This indicates that the kids were maintained under normal health condition without any adverse effects of molasses supplementation on cellular metabolism and liver function as supported by the observed higher serum TP and improved growth performance. However, the significant effect of MC × MD on ALT could be attributed to dietary adaptation. Similarly, it has been concluded that dietary supplementation with urea-molasses mineral granules or urea-molasses mineral blocks had no significant effect on AST and ALT concentrations in kids and adult goats [13, 22, 28].

In the present study, the mean values of Na^+^, K^+^ and Cl^−^ for the control group were within the normal range for goats [25]. The significant changes in the serum Na^+^, K^+^ and Cl^−^ in response to molasses supplementation reflected negatively on SID_3_. Many investigators concluded that the reduction in SID_3_ can be explained by the reduced concentration of strong cations (Na^+^ and K^+^) and/or by the increased concentration of strong anions (Cl^−^) [29]. On the other hand, the present study suggests that the significant increase in serum K^+^ and Cl^−^ reported in the current study accompanied by a significant decrease in blood pH can be attributed to the impaired Na^+^/K^+^ ATPase pump and Na^+^/H^+^ antiporter activity. It has been noted that higher extracellular H^+^ (acidemia) slows membrane Na^+^/H^+^ antiporter activity and thus reduces the cytoplasmic Na^+^ concentration, which in turn depresses K^+^ uptake via Na^+^/K^+^ ATPase pump accompanied by a rapid increase in extracellular Cl^−^ concentration [30].

### Rumen fermentation indices

The range of ruminal pH recorded in the current study was 6.30–6.48. The significant decrease in the ruminal pH in response to dietary molasses supplementation at a concentration of 40% (M-40) in comparison to the other groups can be attributed to the observed significant increase in the ruminal VFAs. The significant decrease in the ruminal pH reported in the current study could also be attributed to the higher sugar and lower fiber contents of molasses. Similar result has been reported by Benavides and Rodriguez [31] who explained the reduction in ruminal pH in sheep fed increasing concentrations of molasses to the decrease in the salivary secretion in response to the lower fiber content of molasses. Interestingly, significant low ruminal pH has been reported previously in kids and adult goats [13, 28], sheep [19] and calves [32] fed urea-molasses mineral granules, urea-molasses blocks, molasses and molasses/concentrate mixture. In contrast, an increase in ruminal pH in bulls and dairy cows fed different concentrations of molasses has also been reported [33, 34].

Dietary molasses supplementation has been reported to increase ruminal VFAs concentration [17, 35], enhanced the supply of amino acids from microbial protein synthesis [13, 36], and gluconeogenesis in animals fed low-quality forage-based diets [37]. In the present study, molasses concentration and the duration of dietary supplementation showed a significant influence on VFAs concentration. The significant increase in VFAs in the supplemented group M-30 and M-40 could be attributed to the rapid and fast degradation of soluble carbohydrates components of molasses. Similar results have been observed by Sahoo et al. [32] who reported that feeding various concentrations of molasses in de-oiled rice bran-based concentrate mixture significantly increased total ruminal VFAs concentration in crossbred cattle calves. Furthermore, Marsetyo et al. [38] noted that total VFAs concentration increased progressively with increasing molasses concentrations in sheep diet. However, the significant increase in VFAs obtained in the present study contradicts the previous reports, which stated that dietary different concentrations of molasses had significantly decreased total ruminal VFAs concentration in bulls and dairy cows [33, 34].

The results obtained in the current study indicated that the duration of dietary molasses supplementation had significant positive impact on total VFAs. The significant decrease in the total VFAs concentration by time can be attributed to the slight higher protozoa count observed. Many investigators explained the presence of ruminal protozoa may contribute in the decrease of VFAs concentration in cattle and buffalos fed molasses and/or urea-molasses cake [33, 39].

The concentration of ruminal NH_3_-N is an effective indicator of ruminal microbes’ activity [40]. The minimum concentration of ruminal NH_3_-N needed for microbial protein synthesis is 5 mg/dL [41, 42], while a range of 10–20 mg/dL is required for an optimum concentration for fiber degradation [43]. The concentration of ruminal NH_3_ reported in the current study exceeded 5 mg/dL and ranged between 7.6 and 10.5 mg/dL. This finding indicates that NH_3_ concentrations observed following molasses supplementation were adequate for optimum microbial growth, and thus efficient rumen fermentation. Many investigators concluded that ruminal NH_3_ concentration more than 5 mg/dL is an effective indicator of ruminal microbes’ activity to support optimum growth in goat kids [44] and goats fed urea-molasses mineral granules and urea-molasses blocks [13, 28].

In the current study, dietary supplementation with higher concentrations of molasses had no significant effect on ruminal NH_3_; however, the duration of dietary supplementation showed a significant effect. This finding contradicts previous reports on goats [28], sheep [19] and cattle [11, 37, 45], which reported a significant decrease in ruminal NH_3_ with increase concentrations of molasses in the diets. Moreover, the significant differences observed by MD and MC × MD, suggested that the ruminal NH_3_ concentration might be associated with the shift of ruminal microbes’ population by time in response to molasses supplementation. This finding indicates the negative impact of both MD and MC × MD on the capacity of ruminal buffering system and energy components (↓K^+^ and ↓VFAs, respectively) observed after 3 weeks. Therefore, the present study recommended the use of molasses for not more than 3 weeks to avoid the negative effect of the interaction between molasses concentration and the duration of dietary supplementation. On the other hand, the numerically slight insignificant higher concentration of ruminal NH_3_ in M-30 indicates the availability of soluble protein in the diet that facilitates the use of excess NH_3_ for microbial protein synthesis. Tuyen et al. [45] reported high and efficient microbial protein production in Brahman crossbred steers fed high-molasses diets (> 50%). Jain et al. [13] explained the significantly higher rate of microbial protein synthesis by the availability of fermentable nitrogen incorporated in microbes’ cells, in addition to keto-acids available from soluble carbohydrates in goat kids fed on de-oiled rice bran supplemented with urea/molasses mineral granules. Furthermore, the results obtained in the present study showed that higher concentration of molasses (45%) resulted in insignificant decrease in ruminal NH_3_ (7.6 mg/dL) compared to the other groups (8.7–10.5 mg/dL). The numerically lower concentration of ruminal NH_3_ in higher molasses supplemented group (M-45) may be attributed to the higher fixation of NH_3_-N as microbial protein in the presence of the observed higher VFAs concentration. Also, it could be due to enhanced ruminal microbes’ growth stimulated by the addition of rapidly fermentable soluble carbohydrates components of molasses. Ciriaco et al. [35] reported a significant decrease in ruminal NH_3_-N concentration due to enhanced ruminal microbial growth in response to dietary molasses in beef steers consuming bermudagrass hay. In lactating dairy cows fed rapidly fermentable sugars, Hristov et al. [46] explained the significant decrease in ruminal NH_3_ by inhibited production of NH_3_, enhanced incorporation of preformed feed amino acids associated with NH_3_ uptake for microbial protein synthesis.

In the present study, dietary molasses supplementation did not show any significant effect on ruminal TPC in goat kids. This finding is consistent with previous results reported by many researchers in different animal species [13, 34]. In contrast, higher ruminal protozoa distribution was reported in Holstein cows fed different concentration of molasses [47]. This increase in ruminal TPC is explained by the higher level of fermentable sugars in molasses, which in turn increases the ruminal concentration of butyrate and consequently, ruminal TPC [33, 47, 48].

The general characteristics of rumen fermentation of goat kids obtained in the current study showed that the rate of energy production in the rumen in the form of VFAs was similar to the rate of NH_3_ released in M-30 until week 3, whereas it was slightly quicker in the other supplemented groups than NH_3_ released. However, ruminal NH_3_ remained at values exceeded 5 mg/dL (8.5–12.5 mg/dL) in all molasses-supplemented groups until week 4, which enhances and facilitates more fixation of NH_3_-N as microbial protein in the presence of the observed higher protozoa population. Several researchers postulated that the efficiency of conversion of NH_3_-N into microbial protein would be maximized if ruminal NH_3_ released at a rate similar to the fermentation rate of the carbohydrate source [33, 48].

In the present study, the changes in the ruminal electrolytes concentrations in response to dietary molasses supplementation reflected on both rumen osmolality and SID_3_. The significant increase in the rumen osmolality is attributed mainly to the observed significant increase in ruminal-[Cl^−^]. Moreover, the significant increase in the ruminal K^+^ (with time) and Cl^−^ (with increasing molasses concentration) in the supplemented groups accompanied by a significant decrease in blood pH might have impaired the Na^+^/K^+^ ATPase pump and Na^+^/H^+^ antiporter activity in the rumen, and consequently resulted in the observed elevated rumen osmolality and reduced SID_3_.

## Conclusion

The results of the current study indicated that dietary supplementation with molasses at a concentration of 30%, and for a duration of 3 weeks had improved growth performance, protein metabolism and resulted in efficient rumen microbial fermentation without compromising animal health, immunity, and electrolytes and acid-base homeostasis in goat kids. However, dietary higher concentrations of molasses (40 and 45%) and longer feeding duration (> 3 weeks) had a slight negative impact on electrolytes and acid-base homeostasis. Therefore, we do recommend dietary inclusion of molasses for up to 30% up to 3 weeks in feeding practices for goat kids fattening.

## Methods

### Ethical approval

All experimental procedures (animal care, sampling) were conducted in accordance with the standards established by the Faculty Research Ethics Committee at Faculty of Veterinary Medicine, University of Khartoum, Sudan. Animal experimentation in the study was approved by Faculty of Veterinary Medicine Research Board (2/2009/5).

### Experimental animals

Twenty clinically healthy male Nubian goat kids (4–6 months old; 9–10 kg BW) were used in the current study. The animals were purchased from Alzareeba livestock local market (Khartoum North, Sudan) based on their body weight. The kids were allowed an adaptation period of 2 weeks, during which the kids were injected with prophylactic doses of broad spectrum antibiotic Oxytetracycline (1 mL I/M for 5 days) and treated against external and internal parasites using Ivermectin injection (1 mL S/C) and Albendazole (5 mL orally), respectively. The kids were then randomly assigned to 4 groups receiving increase concentration of molasses: 0% (M-0), 30% (M-30), 40% (M-40) and 45% (M-45) for a period of 5 weeks. At the end of this study (after week 6), 3 kids of each group were fistulized to be used for another study to determine the effects of dietary supplementation with different concentrations of molasses on fibre digestibility (not reported in the current study). The rest of the kids were fed the basal diet with free access to fresh drinking water. Then the animals were transported to the local market for selling.

#### Housing and management

The kids were housed individually in shaded corral pens made of concrete floor, zinc roof and wire net side (1.5 m × 1.5 m × 2 m). The pens were sprayed with 10% sypermethrin as antiparasitic agent and 40% formalin as a disinfectant.

#### Chemical analysis of the feed

Four diets were formulated according to the standard nutritional requirements of goats [49]. Fresh *Sorghum biocolor* (L.) (Abu 70) was dried under shade, grinded and added to the concentrate in equal proportion to formulate the experimental diets. The diets ingredients were mixed manually until a final uniform mash mixture was obtained. The ingredients and the chemical composition of the experimental diets are shown in Tables 4 and 5. The chemical composition of the experimental diets was estimated on dry matter basis. The analysis was performed according to the procedure described by the Association of Official Analytical Chemists [50].

**Table 4:** Ingredients composition of the experimental diets

Ingredients (%)	M-0	M-30	M-40	M-45
^a^ Sorghum	52	22	22	22
Wheat bran	25	15	10	7
Groundnut cake	9	18	13	11
Groundnut hull	12	13	13	13
Molasses	0	30	40	45
NaCl	1	1	1	1
Limestone	1	1	1	1
Total	100%	100%	100%	100%

*M-0* (0% molasses), *M-30* (30% molasses), *M-40* (40% molasses) and *M-45* (45% molasses)

^a^*Crushed grains + dried Sorghum biocolor (L.)*

**Table 5:** Chemical analysis of the experimental diets

Ingredients (%)	M-0	M-30	M-40	M-45
DM (%)	93.2	89.8	88	88
Fat (%)	2.5	1.7	0.6	0.6
CP (%)	25.5	26.5	25	25
CF (%)	10.1	12.3	9.4	9.4
Ash (%)	6.9	9.7	3.1	3.1
NFE (%)	48	50	32	32
ME (Kcal)	1.1	1.0	1.0	1.0

*DM* Dry matter, *CP* Crude protein, *CF* Crude fibre, *NFE* Nitrogen-free extract, *ME* Metabolizable energy, *M-0* (0% molasses), *M-30* (30% molasses), *M-40* (40% molasses) and *M-45* (45% molasses)

Molasses used in the current study was purchased from Sennar Sugar Factory (Sennar City, Sudan), and composed of sugars (14% glucose, 32% sucrose and 16% fructose), and non-sugars (10% azotic material, 0.5% SiO_2_, 3.5% KO_2_, 1.5% CaO, 0.1 MgO, 0.2% P_2_O_2_, 0.2% MnO_3_, 1.6% SO_3_, 0.4% chloride, 10.5% Ash and 20% water) [51]. Molasses was selected as an available, cheap and energy rich feed stuff.

#### Measurement of daily feed and water intake

Each kid was offered 0.5 kg of the experimental diet daily and the refusal was weighed in the next day and subtracted from 0.5 kg to estimate daily feed intake. Three litres of fresh water were offered for each kid daily, and the rest water was measured in the next day using a measuring cylinder to estimate daily water intake.

#### Growth performance and feed conversation ratio

The initial body weights were recorded on the first day of the experiment at 9:00 am before the morning feeding to avoid weighing errors due to the variation in gut filling. Then the kids were weighed weekly using a traditional balance (Every, UK). Average daily gain (ADG) and feed conversion ratio (FCR) were calculated using the following equations:

$$ ADG\ \left( kg/ day\right)=\frac{Final\ body\ weight- Initial\ body\ weight}{5\  weeks\ } $$

$$ FCR=\frac{Total\ Feed\ intake}{Body\ weight\ gain} $$

### Sample collection

#### Blood collection

The blood samples were collected weekly before the morning feeding by jugular venipuncture using plastic syringes. A 2 mL of blood sample was transferred immediately into EDTA vacutainer tubes, and used to determine the blood pH (HANNA instruments pH meter, Portugal), and for hematological analysis. Another 3 mL was transferred immediately into non-heparinized vacutainer tubes, which was allowed to clot at room temperature and centrifuged (Gallenkamp Junior centrifuge, UK) at 3000 rpm for 15 min. Thereafter, serum samples were collected into sterile Eppendorf tubes and frozen at − 20 °C for further analysis.

#### Rumen liquor

The rumen liquor samples were collected weekly before the morning feeding using a rumenocentesis technique [52]. The kids were restrained without sedation or anesthesia. The puncture site was located 12–15 cm caudal to the costochondral junction of the last rib, on a horizontal line level with the top of the patella. Before rumenocentesis, the puncture site in the left flank was shaved, disinfected with 70% Ethanol and locally anesthetized. The puncture was performed using a 15 gauge, 1½ inch hypodermic needle attached to 20 mL disposable plastic syringe, which was inserted through the skin into the ventral ruminal sac. A volume of 15 mL of the ruminal fluid was aspirated slowly to avoid the changes in the pH of the sample. Then 3 mL of rumen liquor was filtered through a mesh-cloth and pipetted into clean dry test tubes where rumen pH was measured immediately (HANNA instruments pH meter, Portugal); the rest of the rumen liquor samples were used to determine TPC. The remaining rumen liquor was acidified by adding 10 mL of rumen liquor to 10 ml of 0.1% N hydrochloric acid and allowed to settle at room temperature for 1–2 h and centrifuged (Gallenkamp Junior centrifuge, UK) at 3000 rpm for 15 min. Then the samples were pipetted in clean dry Eppendorf tubes and frozen at − 20 °C for further analysis of NH_3_ and VFAs.

### Laboratory analysis

#### Hematological parameters

The blood Hb, HCT and TLC were determined by standard hematological techniques [53]. Blood-[Hb] in g/dL was determined by the Cyano-methemoglobin method. The HCT was determined as percentages by the micro-hematocrit method using capillary tubes and hematocrit reader. The capillary tubes were filled with the blood to about 3/4 and one end was sealed by cristaseal. Then the tubes were centrifuged at 12000 rpm for 5 min in a micro-hematocrit centrifuge (Hawksley, London). The TLC (× 10^**3**^/μL) was counted manually using the improved Neubauer hemocytometer (Hawksly and Sons, Ltd., England), Turk’s solution as a dilution fluid (glacial acetic acid 1 mL, 1% aqueous gentian violet 1 mL and distilled water up to 200 mL), and a light microscope under low power objective (× 10) (Olympus Optical Co, Ltd., Japan).

#### Serum-[TP], [albumin], [AST] and [ALT]

Serum TP and albumin were determined using Biuret and Bromcresol green standard spectrophotometric methods [54, 55] using commercial kits (Spinreact, Spain). The values of TP and albumin were used to calculate serum globulins and A/G ratio. Serum AST and ALT were determined using standard spectrophotometric methods [56] and commercial kits (Spinreact, Spain).

#### Serum electrolytes and the calculation of SID_3_

Serum Na^+^ and K^+^ were determined using a flame photometer technique (PFP7 Jeway, EU), whereas Cl^−^ was determined using a spectrophotometric method and commercial kits (Spinreact, Spain). The values of electrolytes were used to calculate SID_**3**_ = [Na^+^] + [K^+^]-[Cl^−^] mmol/L [29].

#### Ruminal volatile fatty acids (VFAs), NH_3_ and osmolality

The ruminal VFAs, NH_3_ and osmolality were determined using a steam distillation [57] Conway units [58] and a freezing point depression Osmometer (OSMOMAT 030, gonotec, Germany), respectively. In the present study, unfortunately, we didn’t analyze the individual VFAs due to the lack of analyzing facility.

#### Total Protozoa count

One volume of rumen contents were fixed and stained with 1 volume of methyl-blue formalin saline solution (100 mL formaldehyde 35%, 900 mL distilled water, methyl-blue 0.6 g and sodium chloride 0.8 g). Then the mixture was stocked in a dark place until the examination. After gentle mixing of fixed rumen liquor sample, one drop was poured on a hemocytometer slide and covered with a cover slip and examined under low power objective (× 10) using a light microscope (Olympus Optical Co, LTD, Japan).

#### Statistical analysis

The sample size calculation for this study was established using the Resource Equation Approach [59, 60]. Statistical analysis was performed using SPSS for Windows version 20. The normal distribution of the individual data was determined using a One-Sample Kolmogorov-Smirnov adjustment test. General linear Model (GLM) ANOVA (Levine’s and Post Hoc Tests) with repeated measurements was used to assess the possible significant differences among the groups and the interaction between molasses concentration and the duration of dietary molasses. The differences among means were determined using Duncan multiple range test. The mean difference was considered significant at *P* ≤ 0.05.

## Data Availability

The datasets and materials used and analyzed during the current study are available from the corresponding author on reasonable request.

## Abbreviations

MC: Molasses concentration
MD: The duration of dietary molasses
MC × MD: The interaction between molasses concentration and the duration of dietary molasses
DFI and DWI: Daily feed and water intake
BW: Body weight
ADG: Average daily gain
FCR: Feed conversion ratio
Hb: Hemoglobin
HCT: Blood Hematocrit
TLC: Total leukocytes count
TP: Serum total proteins
AST: Aspartate transaminase
ALT: Alanine transaminase
[Na^+^], [K^+^], and [Cl^−^]: Sodium, potassium and chloride ions concentration
SID_3_: Strong ion difference
[NH_3_]: Ammonia concentration
VFAs: Volatile fatty acids
TPC: Total protozoa count
